# Improving Depressive Symptoms through Personalised Exercise and Activation (IDEA): Study Protocol for a Randomised Controlled Trial

**DOI:** 10.3390/ijerph18126306

**Published:** 2021-06-10

**Authors:** Aitana García-Estela, Natalia Angarita-Osorio, Sandra Alonso, Maria Polo, Maria Roldán-Berengué, Monique Messaggi-Sartor, Estanislao Mur-Mila, Laura Vargas-Puertolas, Víctor Pérez, Esther Duarte, Francesc Colom

**Affiliations:** 1 Mental Health Research Group, Hospital del Mar Medical Research Institute (IMIM), 08003 Barcelona, Spain; agarcia6@imim.es (A.G.-E.); nangarita@imim.es (N.A.-O.); 62891@parcdesalutmar.cat (M.R.-B.); emur@parcdesalutmar.cat (E.M.-M.); 61155@parcdesalutmar.cat (V.P.); 2 Department of Psychiatry and Forensic Medicine, Faculty of Medicine, Autonomous University of Barcelona, 08193 Barcelona, Spain; 3 Department of Medicine, Faculty of Medicine, Autonomous University of Barcelona, 08193 Barcelona, Spain; Sandra.Alonso.Marsol@uab.cat (S.A.); mmessaggi@imim.es (M.M.-S.); eduarte@parcdesalutmar.cat (E.D.); 4 Physical Medicine and Rehabilitation Department, Hospital del Mar, Parc de Salut Mar, 08003 Barcelona, Spain; 5 Institute of Neuropsychiatry and Addictions, Hospital del Mar, Parc de Salut Mar, 08003 Barcelona, Spain; mpolo@parcdesalutmar.cat (M.P.); 64306@parcdesalutmar.cat (L.V.-P.); 6 Rehabilitation Research Group, Hospital del Mar Medical Research Institute (IMIM), 08003 Barcelona, Spain; 7 Centre for Biomedical Research in Mental Health Network (CIBERSAM), 28029 Madrid, Spain; 8 Department of Basic, Evolutive and Education Psychology, Faculty of Psychology, Autonomous University of Barcelona, 08193 Barcelona, Spain

**Keywords:** depressive symptoms, exercise, personalised medicine, blended intervention, transdisciplinary

## Abstract

Individuals who suffer from depressive symptoms experience a substantial impact on psychosocial functioning, physical health, mortality, and quality of life. In the search for therapeutic strategies, exercise has been found to play a relevant part in its treatment. However, the promotion of exercise entails adherence difficulties that arose out of the tendency towards sedentarism led by symptomatology. Personalised exercise plans on top of usual care have the potential to enhance behavioural changes and mental health. The present study aims at evaluating the changes in functioning deriving from a blended intervention merging a psychological intervention with a personalised exercise programme based on medical assessment. We will conduct a three-arm randomised controlled trial in which 172 participants suffering from mild–moderate depressive symptoms will be allocated to Intervention A (personalised exercise group programme + app with motivational messages), B (personalised exercise group programme + app with no motivational messages) or control group (app with no motivational messages). Data regarding global functioning, well-being, symptoms, physical activity, and exercise capacity will be collected at baseline, 4, 12, and 36 weeks. The results of this trial will provide information about whether this physical activity support programme may be efficient for improving mental and physical health outcomes. Trial registration: ClinicalTrials.gov NCT04857944 (accessed on 15 April 2021). Registered April 2021.

## 1. Introduction

Major depressive disorder (MDD) was reported as the third leading cause of years lived with disability in the world and the third leading cause in the areas with the highest sociodemographic index by the Global Burden of Disease [1]. Individuals presenting depressive symptomatology experience, regardless of their diagnoses, a significant impact on social and work functioning, physical health and mortality.

Moreover, despite the wide range of effective treatments licensed for depression, MDD is the first worldwide leading cause of impairment and it is globally suffered by over 300 million people [2]. This is partly due to undertreatment being the rule rather than the exception for depressed patients. In high-income countries, only one in five depressed individuals receives systematically minimally adequate treatment [3].

A symptom-based, dimensional view of mental health disorders has the practical advantage of genuinely respecting individual differences. By lacking rigid nosotaxis, professionals are free to enrich each patient clinical case description and their derived needs. One-size-fits-all categorical descriptions lead too often to guideline/algorithm diagnostic-driven therapeutic recommendations which most of the time may not cover patient needs and will not consider patient-already-existing capabilities. In other words, diagnostic-driven treatment algorithms too often become a Procrustes bed: nobody ever fits therapeutic recommendations, which usually end up becoming impractical.

Conversely, the very intrinsic nature of symptom-based approaches—per se respecting individual differences—enhances personalised medicine. The symptom-based mental-health paradigm becomes, by definition, transdiagnostic, a word that describes most daily clinical jobs. The path to precision medicine in mental health is being built and various transdiagnostic treatment programmes for affective disorders are being designed [4,5].

A solid body of scientific evidence supports the regular practise of exercise and validates the many benefits it brings for people with mental disorders, among which are the reductions of depressive symptoms, a positive anxiolytic effect and better quality of life [6]. In support of this, structured and limited in time exercise programmes are particularly recommended as treatments for people with mild to moderate depression or with persistent subthreshold depressive symptoms [7].

However, the effective promotion of this lifestyle entails a challenge, essentially due to adherence difficulties arising out of the tendency towards sedentarism usually led by symptomatology (apathy, fatigue, low motivation, anxiety, etc.). People with MDD engage in high levels of sedentary behaviour and low levels of physical activity [8], which are predictors of mortality. Moreover, higher levels of symptoms of anxiety are associated with higher levels of sedentary behaviour [9].

Here, special consideration must be given to what seems the most important factor associated with the performance of regular exercise among the population suffering from mood disorders: the delivery of advice or indications by health professionals [10]. Personalised medicine stresses a practical need for transdisciplinary effort.

Physical Medicine and Rehabilitation Therapy can have a positive impact on patients with depressive symptoms. Specialists in this field are uniquely positioned to evaluate a patient’s current physical capabilities and limitations, design and discuss a personalised treatment plan and prescribe tailored exercise routines to effectively cope with depressive symptoms in a realistic approach.

Group exercise sessions have specifically shown great benefits [11], and beyond physiological changes and the regulation of sleep produced by physical activity per se, this modality represents added value as it boosts social contact, mirroring, interaction and facilitates the support of other people. Furthermore, the implementation of group interventions is proposed as an alternative to maximizing the optimisation of the use of available resources and services.

A multidisciplinary-based approach merging Physical Medicine and Rehabilitation care with psychological interventions focusing on needs and capabilities, and tailored exercise routines should have an impact on motivation to increase physical activity—including both aerobic and anaerobic exercises—and enhance behavioural changes towards a healthier lifestyle.

We hypothesized that participation in a brief app-blended group intervention promoting personalised exercise and activity, as an add-on to treatment-as-usual, will improve functioning and well-being. Both efficacy measures would result in a lesser impact of depressive symptomatology in life.

The main objective of this research is to evaluate the changes in functioning deriving from a blended intervention merging psychological intervention aimed at increasing activity and exercise with a personalised exercise programme based on medical assessment on subjects suffering from mild to moderate depressive symptoms.

## 2. Materials and Methods

### 2.1. Study Design

We will conduct a 3-arm randomised controlled clinical trial in which 172 eligible participants will be allocated to one of the following conditions:

- Intervention A (*N* = 48): Personalised exercise group programme + smart band + IDEApp (Mass Factory Urban Accessible Mobility S.L, Barcelona, Spain) with motivational messages: After study entry and baseline assessments, subjects will attend the one-month IDEA group sessions aimed at promoting physical activity and exercise. Participants randomly assigned to this study arm will use the smart band and IDEApp with the motivation set enabled, allowing participants to receive the messages according to their compliance and adherence to the personalised prescriptions. After group sessions, study subjects will start receiving the messages in Week 4, and up until the end of the trial. Intervention A study subjects will be expected to continue using the smart band and IDEApp for 8 consecutive months.
- Intervention B (*N* = 48): Personalised exercise group programme + smart band + IDEApp without motivational messages: Subjects will follow the same procedure as Intervention A, with the difference that the IDEApp will have the motivation set disabled and therefore will not receive any messages regarding their compliance. After group sessions, study subjects will be expected to continue using the smart band and IDEApp for 8 consecutive months.
- Control Group (*N* = 76): smart band + IDEApp without motivational messages: After study entry and baseline assessments, all patients assigned to the control group will receive both the IDEApp and the smart band, but the motivation set will be disabled. Study subjects will be expected to use the smart band and IDEApp for 8 consecutive months.

All participants, regardless of their treatment condition, will be read a short text about the benefits of regular exercise on mood during the initial clinical interview, in order to make sure that they have listened to this unspecific advice at least once.

**Figure 1:**
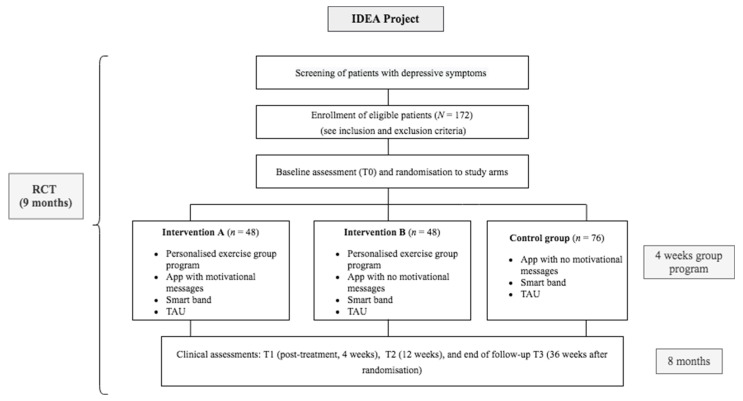
Flowchart of the IDEA Project. IDEA: Improving Depression through Exercise and Activity; RCT: randomised controlled trial; TAU = treatment as usual; T1 = 4-week assessment; T2 = 12-week assessment; T3 = 36-week final assessment.

All participants will continue receiving naturalistic pharmacological and/or psychological treatment, without any research-related disruption. A schematic diagram including the schedule of enrolment, assessments, and visits for participants is available in Figure 1.

We hereby propose a seminal project on the efficacy of personalised exercise plans and psychological advice leading to a regular activity increase on top of the usual treatment regime of subjects presenting with depressive symptomatology: The IDEA (acronym standing for “Improving Depression through Exercise and Activity”) programme. Moreover, the proposal also introduces the advantages of e-mental health to maximise the results. The inclusion of a digital platform app format as a mobile support ‘companion’ tool and a smart band in the project has the potential to prolong motivation and exercise performance and maintain the benefits in the long term. At the same time, it allows for a non-invasive and fine-tuning measuring of variables and changes related to the activity.

### 2.2. Study Setting

The research setting will be outpatient mental health centres and General Practice surgeries belonging to the healthcare network of Parc de Salut Mar in Barcelona, Spain, which covers a catchment area of over 700,000 people for mental health. The available Gross Domestic Product of this area is below average when compared to other districts of Barcelona city. In addition, this area includes some of the neighbourhoods with the lowest incomes in the city [12].

### 2.3. Eligibility Criteria

All participants will meet the following inclusion criteria: aged 18–65 years, presenting mild to moderate depressive symptoms according to the Montgomery–Asberg Depression Rating Scale (MADRS score > 16 and <34), currently owning an Android-compatible smartphone, fluent in Spanish language, basic knowledge and skills using a smartphone, and able to provide written informed consent to participate. Potential participants will be excluded as per the following exclusion criteria: severe cognitive and/or physical impairment; cognitive deficit or developmental disorder; current psychotic, melancholic or catatonic futures; drug or alcohol dependence; modification of drug treatment (or its dose) in the last month; beginning of psychological treatment in the last month; beginning of biophysical treatment in the last month; BMI > 40; physical disability. Any general practitioner, nurse, psychologist or psychiatrist treating the patient will make the referral.

Group sessions will be facilitated by a psychologist (Psychology BSc, MSc) and a physiotherapist (Physiotherapy BSc, MSc) with accredited experience who will receive a brief therapy training.

Before any study procedures occur, participants will be informed about the study characteristics, they will be provided with a study information leaflet and a written informed consent form must be signed by them. After signing the informed consent, an experienced (more than five years of regular clinical practise since specialisation) clinical psychologist or a psychiatrist will confirm or disregard depressive symptomatology, administer the MADRS and judge that the subjects fulfil all the inclusion criteria items and none of the exclusion criteria requirements.

### 2.4. Interventions

#### 2.4.1. Intervention Description

The main goal of the intervention is to personalise exercise prescription and enhance motivation towards being physically active. To fulfil our objectives, a collaborative team of 4 psychologists, 2 Physical Medicine and Rehabilitation specialists (one medical doctor specialised in the field and one physiotherapist), and 1 psychiatrist designed a brief group intervention—the IDEA programme.

The programme will consist of six 90 min group sessions composed of four to six participants as working with small groups facilitates the patient’s integration and allows professionals to better detect their needs. These short intervention sessions will be distributed in one month, with a frequency of once or twice a week depending on the week of the programme. Group sessions will take place in the therapy room of Centre Fòrum–Parc de Salut Mar, which provides rehabilitation, sociosanitary and mental health services. In the exercise-oriented sessions, participants will be asked to wear sports clothes to practise the individualised exercise prescription on-site and will receive an individual brochure with indications and photographs of their prescription.

For the implementation of the sessions, we created a guide for the professionals where the content of each session and its dynamics were outlined. In the case of the individualised exercise prescription, a pool of exercises was created in order to standardise the potential exercises that will be chosen individually for each participant. Table 1 presents an overview of the IDEA programme. Detailed information regarding the content of the group sessions is available in Table S1 as Supplementary Material.

The exercise prescription will be personalised in terms of intensity and amount of time. Therefore, all participants will perform four types of exercise: stretching, aerobic, strength and relaxation. We have designed three types of programmes covering low, moderate and high intensity. Participants with higher levels of sedentarism will be prescribed 45 min sessions twice a week, while participants with lower levels will do 60 min sessions three times a week.

Participants will choose the type of aerobic exercise according to their preferences and possibilities (i.e., having easy access and knowing how to perform the exercise). Options can include—but are not limited to—walking, running, water aerobics, biking or dancing.

The intensity of the aerobic exercise will be based on the maximum age-related heart rate estimated by subtracting the age of participants from 220. All participants will receive instructions on how to keep track of their heart rate. In the low and moderate intensity programmes, participants should keep their heart rate between 45 and 54% of their maximum heart rate, while participants receiving the high-intensity programme should keep a heart between 70 and 89%. Further information regarding the personalised exercise prescription is detailed in Table S2 contained in the Supplementary Material.

As a result of the COVID-19 pandemic, we are currently facing many uncertainties around this rapidly evolving situation and the governmental measures responding to it. The IDEA trial will be conducted undertaking risk assessments and adapting trial processes when necessary. The intervention will be delivered ensuring participants and professionals’ safety by following the advice of local public health authorities (e.g., mandatory use of hygienic or surgical masks, participants will be encouraged to maintain physical distance, fitness material used will not be shared during group sessions and will be properly disinfected before and after the sessions, doors will remain open and alcohol-based hand rub will be at participants’ disposal).

#### 2.4.2. Application Development

Simultaneously, the same working group that designed the intervention designed the application describing the characteristics of an app meant both to store activity and sleep data and able to send motivational messages to the user whenever an activity decline or sleep problem was identified. The group designed the algorithms thought to classify adherence or lack of it regarding physiotherapist prescriptions. After putting the software development out to tender—a must, as the project is receiving public funding—the selected app developer software company (Mass Factory Urban Accessible Mobility S.L., Barcelona, Spain) was contacted and the team started a co-creative process including several software engineers and designers to create an ad-hoc-made app both to register and store the participants’ physical activity, exercise compliance and sleep patterns and to send the already mentioned motivational messages when needed.

The result of this process was a user-friendly smartphone application—IDEApp—that can be synchronised to a smart band. The smart band will register, store data regarding physical activity (i.e., heart rate) and sleep patterns, as well as provide information about daily exercise practice. IDEApp will collect two types of data: objective and self-informed. Objective data will include the number of daily steps, aerobic exercise—including minutes, distance in metres, and maximum, minimum and average heart rate—and sleep structure, including duration in a 24-h period, and deep and light sleep discrimination. On the other hand, self-reported data will require participants to simply indicate on IDEApp whether they have performed one of three exercise options: (1) relaxation, (2) stretching, or (3) strength and endurance.

**Figure 2:**
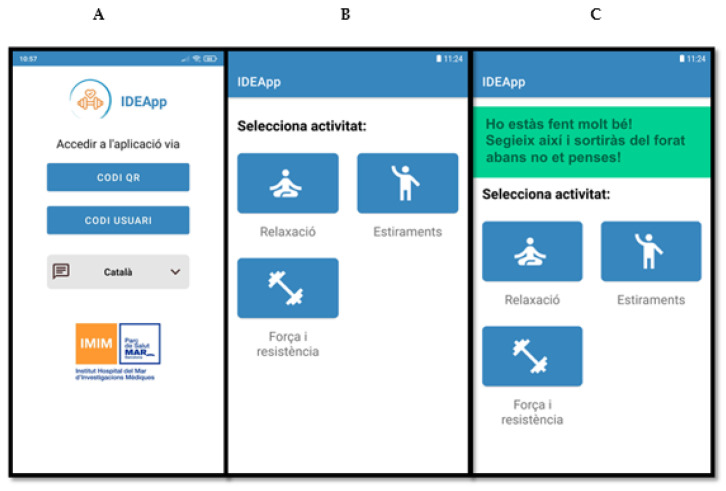
Screenshots of IDEApp running on a smartphone (messages in Catalan). (**A**)—login and register screen; (**B**)—home Screen without notification; (**C**)—home screen with motivational notification.

When non-compliance with the exercise prescription, physical activity decreases, or altered sleep patterns are detected, the system will trigger motivational or awareness messages through the app. The motivational messages were classified according to 12 potential situations following an algorithm based on the percentage of exercise prescription performed and the number of hours of sleep. A pool of more than 50 motivational messages was created for each category. Detailed information about the algorithm and type of messages is displayed in Table S3 presented in Supplementary Material. Accessibility options of IDEApp include features relating to language (Spanish or Catalan) and text size. Screenshots of IDEApp running can be found in Figure 2. Messages’ function will be “on” for only one of the trial arms and “off” for the rest of subjects. The app was named “IDEApp” for obvious reasons.

#### 2.4.3. Criteria for Discontinuing or Modifying Allocated Interventions

Participants may withdraw from the study for any reason at any time. The investigators also may withdraw participants from the study in order to protect their safety and/or if they are unwilling or unable to comply with required study procedures (i.e., health care providers notice a serious adverse event including hypomania or mixed features).

#### 2.4.4. Strategies to Improve Adherence to Interventions

The inclusion of a mobile support tool (IDEApp) that delivers motivational messages and a smart band in the trial is not a core part of the intervention but, rather, a way to monitor activity and sleep patterns, and a strategy to improve adherence to the intervention, which has the potential to prolong motivation and exercise performance in the long term. All participants will benefit from monitoring their exercise physical activity and sleep patterns, regardless of their allocation to study condition. In addition, IDEApp will deliver motivational messages to participants allocated to Intervention A when the smart band detects activity decrease, non-compliance with the exercise prescription or strange sleep patterns.

#### 2.4.5. Relevant Concomitant Care Permitted or Prohibited during the Trial

This study seeks to investigate the effects of personalised exercise in addition to standard of care. It tests a trans-paradigmatic intervention that does not require therapist—or user—to strongly adhere to—or renounce—ideological models of mental health. It is fully compatible with other psychological therapies. All concomitant care and interventions are permitted if they were implemented more than a month before study entry.

### 2.5. Outcomes

#### 2.5.1. Primary Outcome Measure

Given the great heterogeneity expected in the sample of patients, the intervention is aimed at improving global functioning in the first place. The functional impairment will be assessed using the 36-item Short-Form Health Survey (SF-36v2, Quality Metric Inc., Lincoln, NE, USA) [13,14]. As the main target of the trial is to learn about the efficacy of personalised exercise prescription, the main outcome comparison will be (Intervention A + Intervention B) vs. Control, being the comparison of A vs. B vs. C considered as secondary.

#### 2.5.2. Secondary Outcome Measures

All the Secondary outcomes will compare both (A + B) vs. C and A vs. B vs. C.

- Depressive symptoms using the Patient Health Questionnaire (PHQ-9) [15,16].
- Well-being measured by the World Health Organization Well-Being Index (WHO-5 WBI) [17,18].
- Motivation for exercise using the Spanish validation of the Exercise Motivations Inventory (EMI-2) [19,20].
- To identify their physical needs and capabilities, we will measure:
- Current physical activity using the Simple Physical Activity Questionnaire (SIMPAQ) [21].
- Functional exercise capacity using the 6 min walking test (6MWT) [22]. The change in the distance walked in the 6MWT after completion of the programme will be used to trace the natural history of change in exercise capacity over time. Before the test, a finger pulse oximeter will be attached to participants to record baseline and final heart rate and oxygen saturation. For the evaluation of perceived exertion, the short Borg CR 10 Scale [23] will be used before and after the test. This modified Borg scale has a score range from 0 to 10 and seems more appropriate than the longer Borg 15 Graded Category Scale, which requires a greater differentiation capacity. The Borg CR 10 Scale showed moderate reliability for patients with depressive and anxiety disorders [24].
- Functional exercise capacities using the 1 min sit-to-stand test [25].
- Isometric muscle strength of the hand and the forearm using the handgrip strength test [26]. The equipment used will be a digital hand dynamometer (JAMAR^®^, Nottinghamshire, UK) [27], and grip strength will be measured 3 times per hand in the 2-handle position.

For all the questionnaires, we will be using the validated versions in the Spanish language.

### 2.6. Sample Size

Initially, we computed a sample size of 152, given a power of 0.8, an alpha level of 0.05 and inferring functioning according to clinical remission data [28]. To our knowledge, functioning as a main outcome for physical exercise in depression has not set a precedent to guide our calculations. Thus, we computed a Cohen’s d effect size of 0.5 to make estimations more reliable. An expected drop-out rate of 20% was set, which resulted according to the initial design in two arms of 76 subjects each. However, during the cocreative process of the intervention, it was discussed whether motivational messages of the app would mask the effect of the group sessions on the outcomes of the study. Therefore, it was decided to include a third study arm to assess the intervention without motivational elements triggered by the app. We increased the sample size by adding 20 participants, which will be distributed into the two experimental groups, leaving us with a sample size of 172.

### 2.7. Recruitment

The recruitment period will extend over 12 months and all participants will be given a smart band that will be free to use together with free access to the app after study completion.

The resources and strategies for identifying and recruiting potential subjects will include: (1) Sending a monthly newsletter to clinical staff in adult inpatient and outpatient wards to keep IDEA in mind. (2) Attending regular clinic meetings. (3) Posting leaflets or posters with a brief overview of IDEA in each clinic room at the trust. (4) Keeping in close contact with clinicians in the trust (outpatient, inpatient, and primary services) to make sure everyone has ongoing awareness and knowledge of the process. (5) Giving an incentive for trainee clinicians such as research ‘tokens’ for psychiatry training portfolio depending on the number of patients referred.

### 2.8. Assignment of Interventions: Allocation

During the baseline assessment, participants will be registered individually in the IDEApp web-based platform, which will randomly assign them to one of the three conditions while maintaining the following distribution:

- Intervention A: participants receiving the intervention and motivational messages (48 participants; 27.9% of the sample).
- Intervention B: participants receiving the intervention and no motivational messages (48 participants; 27.9% of the sample).
- Control group: using the smart band without motivational messages (76 participants; 44.2% of the sample).

When subjects enter the study, they will be allocated to whichever condition is furthest from the expected percentage of subjects allocated. This allocation system will allow us to guarantee that all groups will have an even distribution at baseline irrespective of the number of participants.

All participants will be identified with a computer-generated random number that will be paired with a 9-digit identification number.

### 2.9. Concealment Mechanism

Allocation will be concealed through the IDEApp web-based system and will be accessible only by request. In order to maintain allocation concealment, only one of the researchers in charge of the evaluation will be able to see the allocation placement. The allocation information will enlighten whether participants have been allocated to the control or intervention condition, but this study researcher will not know which of the experimental conditions (A or B) participants have been allocated to. The purpose of knowing the placement of participants is to inform them whether they must attend the IDEA group sessions or not.

### 2.10. Implementation

Allocation and intervention assignment will be automatically generated by the IDEApp web-based platform once participants are registered in the system by study staff. A study psychologist will enrol participants in the trial after baseline assessment.

### 2.11. Blinding

For logistics purposes, after assignment to study conditions, trial participants will know whether they have been allocated to experimental or control conditions, but the specific experimental intervention (A or B) will not be revealed. Unfortunately, it is hardly achievable to blind therapists and physiotherapists delivering the group sessions. Evaluators blind to treatment condition will carry out baseline assessments. To avoid assessment bias, an independent evaluator blinded to group allocation will complete follow-up assessments. Other care providers (i.e., clinicians), statisticians and principal investigators will be blind to randomisation procedure and group allocation.

Due to ethical considerations, the blinding process will be interrupted if a risk situation is detected during any phase of the study. In this case, the subject will be identified, and their clinician will be informed immediately. Cessation of participation will be discussed depending on the circumstances.

### 2.12. Data Collection and Management

#### 2.12.1. Plans for Assessment and Collection of Outcomes

A trained psychologist (Psychology BSc, MSc) and a physiotherapist (Physiotherapy BSc, MSc) will carry out the assessments. Evaluation instruments have been described in the Outcomes section, and time points and assessment tools are outlined in Table 2.

Following referral, participants will attend the study entry clinical interview and baseline assessment, followed by an evaluation with a physiotherapist to identify their physical needs and capabilities. Such assessments will allow for the subsequent prescription of tailored exercise to the intervention groups. After baseline assessment, the study staff will install IDEApp on the smartphone of all participants and provide them with the smart band. Instructions on how to use both will be provided, alongside written information with basic information and contact information (e.g., help phone or email) where technical issues regarding device use will be resolved. In the first week after study entry, all subjects will effectively start using the devices and IDEApp, which will provide estimates of their baseline physical activity and exercise, and up until the end of the trial by implementing ecological momentary assessment methods (EMA). This monitoring system using a wearable band and a mobile phone-based EMA includes objective measures of physical activity and health variables, such as heart rate, walking and sleep patterns, and is intended to be minimally invasive to the users and their mobile phone usage. The EMA methods are expected to provide with accurate data on activity changes that will allow for adapting motivational messages according to the activity states.

After completion of the group sessions (or after 1 month for Control Group), every participant will be followed-up for eight months. Post-allocation assessments following the intervention at 4 weeks (T1) and 12 weeks (T2) will be carried out by telephone in order to ease the burden of participants. These assessments will last 30 min and will be performed from the facilities of the Centre Fòrum in order to guarantee data safety. The final assessment at 36 weeks (T3) will be carried out face-to-face and will follow a similar outline as the baseline assessment, and again will be carried out by a psychologist and a physiotherapist.

#### 2.12.2. Plans to Promote Participant Retention and Complete Follow-Up

The plans to promote participant retention and complete follow-up include: (1) delivering hand-outs with highlights at the end of each session; (2) contacting participants when no records of the smart band are detected by the IDEApp web-based platform; (3) contacting participants to remind them about the upcoming appointments and let them know what to expect from them and the estimated time frame; (4) availability and flexibility of study stuff to arrange assessment visits at convenient times; (5) developing good therapist–client alliance.

#### 2.12.3. Data Management

Study data will be collected, entered, and managed using REDCap electronic data capture tools hosted at IMIM [30], which offer a free and secure method of robust data collection. More specifically, data will be collected offline in the REDCap mobile app on an Android tablet and then will be sync back to the project on the REDCap server. A password system will be utilised to control access to data and the activity that researchers may undertake is regulated by the privileges associated with their user identification code.

Original study consent forms will be entered, stored in numerical order and kept on file in locked cabinets at the site for a period of 5 years after completion of the study. Access to the study files will be restricted.

#### 2.12.4. Confidentiality

In order to ensure participants’ confidentiality during the study and the transmission of personal data, we will set a security protocol in accordance with the local Spanish laws. A 9-digit identification number (IDN) will be generated for all the participants throughout all the phases of the study. The cross-reference of this identification number and the patient identity will be encrypted and stored in a database file kept in a computer without access to the Internet (neither by wire nor Wi-Fi). Patients will be identified by the IDN, and a random number assigned by the app, which will be the user code to access the application too. Study participants will be requested by a user or QR code when signing into the app. Personal information will not be collected by IDEApp.

All data collected by the smart band and IDEApp will be processed applying technical and organisational security measures established by the current legislation (Organic Law 3/2018, of 5th December, on Protection of Personal Data and guarantee of digital rights). The database will be stored in a physical server kept in the IMIM-Parc de Salut Mar facilities. This information will be used solely and exclusively to carry out analyses related to the objectives of the study and will be stored anonymously in a database during and after the trial.

## 3. Statistical Methods

### 3.1. Statistical Methods for Primary and Secondary Outcomes

Statistical analysis will be performed using the software SPSS Statistics 25 (IBM Corp., Armonk, NY, USA). Descriptive statistics will be used to analyse the distribution of socio-demographic and clinical characteristics among groups at baseline. Continuous variables with a normal distribution will be analysed performing an ANOVA. Where the premises of normalcy are not met, the Wilcoxon test will be used. The differences between groups on the categorical and mail clinical variables will be evaluated by using a Chi-square test. Those variables that are statistically significant may be used as covariates for a logistic or linear regression study of the factors associated with the magnitude of the effect, and determine which variables are better predictors of functioning. The effect size index will be estimated in case of correlation indexes for each of the performed analyses. To analyse efficacy, we will perform intention to treat analysis. Analysis will be two-tailed and the significance set a *p* < 0.05.

### 3.2. Methods in Analysis to Handle Protocol Non-Adherence and Any Statistical Methods to Handle Missing Data

Last Observation Carried Forward analysis will be used to handle and minimise missing data on the clinical variables.

## 4. Ethics and Dissemination

All the procedures and assessments will follow the accordance of the 1964 Helsinki declaration and its following amendments (64th WMA General Assembly, Fortaleza, Brazil, October 2013). Before any procedure, all the participants will be informed about the study characteristics, and all will be provided with a study information leaflet and written informed consent will be signed. The project has been approved by the IMIM Drug Research Ethical Committee (2019/8816/I).

The study will follow the Consolidated Standards of Reporting Trials (CONSORT) guidelines [31] to describe, report and publish the results. The moment the trial is completed, results will be published in international peer-reviewed journals.

## 5. Trial Status

At the time of the elaboration of this manuscript, the development and testing phase of the IDEApp was finalised after being carried out intensively by three software engineers, three psychologists and one physical medicine specialist. Moreover, the test of the smart band was completed, as well as the co-creation of group contents. In order to be timely aware of improvable issues regarding the programme contents and duration, and to identify IDEApp mistakes and bugs, we are running a pilot with subjects who suffer depressive symptoms. Conclusions from this pilot will be used to fine-tune the programme final version and a “Work in progress” manual will be written to ensure research falsifiability.

## 6. Discussion and Conclusions

This project is subject to some limitations. First, participants who exercise regularly prior to the study inclusion may result in a confounding factor. Second, the physical assessment includes Body Mass Index as a measure of obesity to ensure that the morbidly obese are not put at risk by exercising. However, this does not exclude sarcopenic obesity, which includes normal weight, hence normal BMI but low muscle mass percentage. The usage of a body fat calliper and asking for a blood count looking for cholesterol levels would help make sure that people with sarcopenic obesity are not put at risk. Third, we assume that our sample will include subjects with adjustment disorders, including complicated grief where both time from the event and cultural issues play a sensible role regarding improvement. This may emerge as a confounding factor, and we should trust random assignment to handle it. Finally, introducing a new technology such as a wearable device can alter participants’ default behaviour; thus, the measurements may not accurately reflect the activity that they would normally perform under non-experimental conditions.

On the other hand, we introduce novel highlights such as using a transdisciplinary approach, performing the recruitment based on symptoms; the inclusion of personalised medicine, guaranteeing that every participant will receive a tailored prescription of exercise; a transdisciplinary team, joining mental health professionals with physical medicine and rehabilitation specialists; the creation of a brief intervention, comprised of one month to promote adherence; a blended therapy, combining a group intervention with IDEApp and the use of a smart band and promoting a behavioural change that might last longer, and finally evaluating functionality outcomes, providing us with more reliable and ecological outcomes.

## Figures and Tables

**Table 1:** Description of the content of the IDEA group sessions.

Session
1 Depression and exercise
2 Motivation towards exercise
3 Introduction to the exercise prescription
4 Barriers to exercise
5 Review of the exercise on prescription
6 What now? Exercise maintenance

**Table 2:** SPIRIT diagram of the IDEA trial.

	Study Period					
	Enrolment	Allocation	Post-Allocation			Close-Out
Time point	−*t*_1_	*t*_0_	4 Week group sessions	*t*_1_	*t*_2_	*t*_3_
Enrolment:						
Eligibility screen	X					
Informed consent	X					
Allocation		X				
Interventions:						
Intervention A			X	X	X	X
Intervention B			X	X	X	X
Control group				X	X	X
Assessments:						
Sociodemographic data		X				
Health habits and clinical data		X				
SF-36		X		X	X	X
WHO-5 WBI		X		X	X	X
PHQ-9		X		X	X	X
SIMPAQ		X		X	X	X
6 min walking test		X				X
1 min STS test		X				X
Hand grip strength test		X				X
EMI-2		X				
Activity and sleep monitoring		X	X	X	X	X
Delivery of motivational messages				X	X	X

SPIRIT = Standard Protocol items: Recommendations for Interventional Trials [29].; *t*_1_ = pre-allocation; *t*_0_ = baseline assessment; *t*_1_ = post-allocation assessment at 4 weeks; *t*_2_ = post-allocation assessment at 12 weeks; *t*_3_ = final assessment at 36 weeks; SF-36 = 36-item short-form health survey; WHO-5 WBI = world health organization well-being index; PHQ-9 = patient health questionnaire; SIMPAQ = simple physical activity questionnaire; STS = sit-to-stand; EMI-2 = exercise motivations inventory.

## Data Availability

Data sharing not applicable. No new data were created or analysed in this study. Data sharing is not applicable to this article.

## Supplementary Materials

The following are available online at https://www.mdpi.com/article/10.3390/ijerph18126306/s1, Table S1: Description of the IDEA group sessions, Table S2: Personalised exercise prescription programmes, Table S3: Algorithm and type of messages of IDEApp.
