# Supplementary material for: Improving Depressive Symptoms through Personalised Exercise and Activation (IDEA): Study Protocol for a Randomised Controlled Trial
Source: Int J Environ Res Public Health. 2021 Jun 10;18(12):6306. doi: 10.3390/ijerph18126306 (PMC8296117; doi:10.3390/ijerph18126306)
Supplement: Supplementary file 1 [file ijerph-18-06306-s001.zip › ijerph-1218044-supplementary.pdf]

Table S1. Description of the IDEA group sessions.

Session	Personnel	Description
1 Depression and exercise	Psychologist and physiotherapist	- Participants' and psychologists' presentation.<br>- Introduction to the program (how many sessions, duration, basic rules).<br>- Discussion on depressive symptomatology, the relationship between depression and exercise, benefits of exercising, recommendations on how to perform the exercise in a safe manner, how to increase physical activity.<br>- Individual dynamic: the relationship between mood and daily life activities.
2 Motivation towards exercise	Psychologist	- Discussion on what is motivation. What are my motivations? How do my motives relate to things that matter in my daily life?<br>- Dynamic: brainstorming of what are the pros and cons of practising and not practising exercise.<br>- Guided exercise: body scan.
3 Introduction to the exercise prescription	Physiotherapist	- Introduction of the personalised training plan prescription for each patient, explanation on how to use the Borg scale and understand the use of heart rate to evaluate the intensity of the exercises.<br>- In-site guided practise of the prescription.<br>- Guided exercise: mindfulness-based guided walk.
4 Barriers to exercise	Psychologist	- Discussion on which are my barriers towards exercise? barriers in the past, the importance of identifying barriers, how to deal with the barriers.<br>- Dynamic: myths regarding exercise: true or false?<br>- Guided dynamic: the exploration of the breathing spaces.
5 Review of the exercise on prescription	Physiotherapist	- Review of the exercise prescription and Q&As'.<br>- Review possible problems (e.g., fatigue, pain)<br>- Review of the use of the smart band and IDEApp.<br>- In-site guided practise of the prescription.
6 What now? Exercise maintenance	Psychologist and physiotherapist	- Discussion about maintenance strategies, tips on how to be physically active.<br>- Review of what has been done in previous sessions and solving remaining doubts.

Table S2. Personalised exercise prescription programs.

PROGRAM	TYPE OF EXERCISE	FREQUENCY/ TIME	INTENSITY
**GREEN**<br>Low intensity.<br><br>Sessions should last a total of 45 minutes.	Stretching	2x week<br>10 min	Not applicable
	Aerobic exercise:<br>Participant choice:<br>____________<br>(i.e., running, walking at high speed, dancing, biking…)	Daily<br>15 min	Perceived exertion according to Borg's scale: 3-4<br>Maximum heart rate: 45-54%
	Strength exercises:<br>Exercises number: i.e., 3, 4, 5.	2 x week<br>One-two sets of 10-12 repetitions (according to participants abilities).<br>Rest of 30 seconds – 1 minute between sets according to recovery capacity.	Perceived exertion according to Borg's scale: 3-4<br>Maximum heart rate: 45-54%
	Relaxation	2x week<br>10 min	Not applicable
**ORANGE**<br>Moderate intensity.<br>Sessions should last a total of 60 minutes.	Stretching	3x week<br>10 min	Not applicable
	Aerobic exercise:<br>Participant choice:<br>____________<br>(i.e., running, walking at high speed, dancing, biking…)	Daily<br>30 min	Perceived exertion according to Borg's scale: 5-6<br>Maximum heart rate: 55%-69%
	Strength exercises:<br>Exercises number: i.e., 3, 4, 5.	3 x week<br>Two-three sets of 10-15 repetitions (according to participants abilities).<br>Rest of 30 seconds – 1 minute between sets according to recovery capacity	Perceived exertion according to Borg's scale: 3-4<br>Maximum heart rate: 45-54%
	Relaxation	3x week<br>10 min	Not applicable
**RED**<br>Vigorous intensity.<br>Sessions should last a total of 60 minutes.	Stretching	3 x week<br>10 min	Not applicable
	Aerobic exercise:<br>Participant choice:<br>____________<br>(i.e., running, walking at high speed, dancing, biking…)	Daily<br>Between 30 – 40 min	Perceived exertion according to Borg's scale: 7-8<br>Maximum heart rate: 70-89%
	Strength exercises:<br>Exercises number: i.e., 3, 4, 5.	3x week<br>Two-three sets of 10-15 repetitions (according to participants abilities).<br>Rest of 30 seconds – 1 minute between sets according to recovery capacity	Perceived exertion according to Borg's scale: 3-4<br>Maximum heart rate: 45-54%
	Relaxation	3x week<br>10 min	Not applicable

Table S3. Algorithm and type of messages of IDEApp.

% Exercise Prescription	Sleeping Hours	Amount of time necessary	Type of message	Example of message
70-100%	4-10	Required duration of 2 days	A (Confirmation of good response to the prescription)	*"Even if your achievements seem small, they are very necessary"*
50%-69%	4-10	Required duration of 2 days	B (Reinforcement of appropriate behaviour)	*"Nice job! What if we add a couple more steps a day?"*
26-49%	4-10	Required duration of 2 days	C (Moderately non-compliant behaviour change attempt)	*"Remember that it takes time and practise, the important thing is to keep going!"*
<25%	4-10	Required duration of 4 days	D (Non-compliant behaviour change attempt)	*"It seems like you are not exercising … we already know is very difficult to start but remember that it is the best way to recovery"*
70-100%	>10.1	Required duration of 2 days	A+ (Confirmation of good response to the prescription, reducing the hours of sleep)	*"Fantastic! You are moving. Getting a little less sleep will help you recover sooner. Avoid napping if you do"*
50%-69%	>10.1	Required duration of 2 days	B+ (Reinforcement of appropriate behaviour, reducing the hours of sleep)	*"Keep up this pace of activity and get a little less sleep - you are doing great!"*
26-49%	>10.1	Required duration of 2 days	C+ (Moderately non-compliant behaviour change attempt, reducing the hours of sleep)	*"We know that staying active takes effort, but try increasing your activity and reduce the number of hours you sleep. It will feel good!"*
<25%	>10.1	Required duration of 4 days	D+ (Non-compliant behaviour change attempt, reducing the hours of sleep)	*"It seems that your activity has decreased, and your sleeping hours have increased. Try to move a little bit more. If it is costing you too much, maybe you need to contact your doctor and see what is happening"*
NA	<4 (One day)	N/A	E (Asking the causes of insomnia)	*"It looks like you are having trouble sleeping. Remember the rest guidelines".*
NA	<4 (2 ds or more)	N/A	E+ (Exploring hypomania)	*"You seem to need less sleep…and that is not necessarily good news. We recommend you contact your doctor"*

NA	Poor Sleep Quality	Required duration of 3 days	F (Addressing severe insomnia)	*"It looks like you haven't been sleeping in the last couple of days… this problem can worsen your depression. Try to exercise in the morning, not taking a nap, and not drinking coffee. If this doesn't work in three days, you should contact your doctor"*
No signal	No signal	Required duration of 2 days	X (Checking smart band use, the message will be sent to the mobile device as an SMS)	*"You seem to have forgotten to wear the smart band in the last few days. Remember to put it on again"*
